# Neuronal input triggers Ca^2+^ influx through AMPA receptors and voltage‐gated Ca^2+^ channels in oligodendrocytes

**DOI:** 10.1002/glia.23670

**Published:** 2019-07-17

**Authors:** Tara Barron, Jun Hee Kim

**Affiliations:** ^1^ Department of Cellular and Integrative Physiology UT Health San Antonio San Antonio Texas

**Keywords:** AMPA receptor, Ca^2+^ dynamics, neuron–glia interaction, oligodendrocytes, voltage‐gated Ca^2+^ channel

## Abstract

Communication between neurons and developing oligodendrocytes (OLs) leading to OL Ca^2+^ rise is critical for axon myelination and OL development. Here, we investigate signaling factors and sources of Ca^2+^ rise in OLs in the mouse brainstem. Glutamate puff or axon fiber stimulation induces a Ca^2+^ rise in pre‐myelinating OLs, which is primarily mediated by Ca^2+^‐permeable AMPA receptors. During glutamate application, inward currents via AMPA receptors and elevated extracellular K^+^ caused by increased neuronal activity collectively lead to OL depolarization, triggering Ca^2+^ influx via P/Q‐ and L‐type voltage‐gated Ca^2+^ (Ca_v_) channels. Thus, glutamate is a key signaling factor in dynamic communication between neurons and OLs that triggers Ca^2+^ transients via AMPARs and Ca_v_ channels in developing OLs. The results provide a mechanism for OL Ca^2+^ dynamics in response to neuronal input, which has implications for OL development and myelination.

## 1 INTRODUCTION

Oligodendrocytes (OLs) ensheath axons with their myelinating processes, providing protection and maintenance of axonal integrity and enabling saltatory conduction of action potentials. Dynamic interactions between neurons and OLs modulate development of OL lineage cells and myelination to control conduction speed in the central nervous system (CNS; Pajevic, Basser, & Fields, 2014; Sinclair et al., 2017). Recent studies demonstrated that a dynamic Ca^2+^ rise in OLs is associated with myelin sheath refinement in vivo in the zebrafish spinal cord (Baraban, Koudelka, & Lyons, 2018; Krasnow, Ford, Valdivia, Wilson, & Attwell, 2018). Ca^2+^ is an important second messenger that induces cellular processes that can lead to local translation and process extension in OL lineage cells (Baraban et al., 2018; Krasnow et al., 2018; Wake, Lee, & Fields, 2011). However, little is known about signaling factors from active axons that facilitate activity‐dependent neuron‐OL communication leading to Ca^2+^ transients in OLs, as well as the sources of OL Ca^2+^ rise in response to neuronal inputs.

Previous studies identified neuronal glutamate or GABA as potential mediators of neuron‐OL interaction (Bergles, Roberts, Somogyi, & Jahr, 2000; Kukley, Capetillo‐Zarate, & Dietrich, 2007; Lin & Bergles, 2004). In particular, OL lineage cells express Ca^2+^‐permeable or Ca^2+^‐impermeable AMPA receptors (AMPARs) and NMDA receptors, and thus can directly sense the glutamate release from neurons (Berret et al., 2017; Ge et al., 2006; Micu et al., 2016; Patneau, Wright, Winters, Mayer, & Gallo, 1994). Recent studies showed that inhibiting/knocking out neurotransmitter receptors in OLs impairs their response to neuronal activity and leads to alterations in OL development and function (Koudelka et al., 2016; Kougioumtzidou et al., 2017; Mensch et al., 2015), suggesting that glutamate can be an important signal for developing OLs to be able to sense the electrical activity of active axons.

Voltage‐gated Ca^2+^ (Ca_v_) channels are potential indirect effectors of glutamatergic input, because activation of AMPA receptors is able to depolarize and activate voltage‐gated ion channels in OL lineage cells (Berret et al., 2017; Káradóttir, Hamilton, Bakiri, & Attwell, 2008). Previous studies have reported the expression of L‐, N‐, and R‐type Ca_v_ channels and the presence of their currents in OL lineage cells (Berger, Schnitzer, Orkand, & Kettenmann, 1992; Butt, 2006). In NG2+ glial cells, integration of synaptic input resulted in Ca^2+^ signals by recruiting low voltage‐activated R‐type or T‐type Ca_v_ channels (Sun, Matthews, Nicolas, Schoch, & Dietrich, 2016). In addition, high voltage‐activated L‐type Ca_v_ channels are known to modulate OL development and migration (Cheli et al., 2016; Cheli, Santiago González, Spreuer, & Paez, 2015; Paez, Fulton, Colwell, & Campagnoni, 2009). Despite functional implications of Ca_v_ channels in OL lineage cells, there is a lack of evidence for the functional link between glutamatergic inputs and Ca^2+^ influx through Ca_v_ channels in developing OLs beyond the precursor stage.

Here, we investigate the mechanism by which glutamate induces a Ca^2+^ rise in OLs in the medial nucleus of the trapezoid body (MNTB) of the auditory brainstem. The heavily myelinated axon fibers in the MNTB, which are important for ensuring the reliability and temporal fidelity of conduction, undergo activity‐dependent myelination during development (Kim, Renden, & von Gersdorff, 2013; Kim, Turkington, Kushmerick, & Kim, 2013; Sinclair et al., 2017). In the MNTB, we find glutamatergic input‐induced Ca^2+^ transients and Ca^2+^ currents via Ca_v_ channels in developing OLs using electrophysiology and Ca^2+^ imaging in mice expressing OL‐specific GCaMP6f, a genetically encoded Ca^2+^ indicator with fast kinetics (Chen et al., 2013). We identify AMPARs and L‐type and P/Q‐type Ca_v_ channels as crucial sources of Ca^2+^ influx in pre‐myelinating OLs in response to glutamatergic neuronal input, coordinating active communication between neurons and OLs.

## 2 MATERIALS AND METHODS

### 2.1 Animals

Experiments using transgenic mice were approved and performed in accordance with the guidelines and protocols of the University of Texas Health Science Center at San Antonio (UTHSCSA) Institutional Animal Care and Use Committee (IACUC, approved protocol #14005×). Heterozygous CNPase‐Cre mice, generated by Dr. Klaus Nave (Max Planck Institute of Experimental Medicine, Germany) and obtained from Dr. Manzoor Bhat (UTHSCSA), were bred with homozygote Cre‐dependent GCAMP6f mice (Ai95D, JAX #028865) obtained from Dr. Martin Paukert (UTHSCSA). Heterozygote CNPase‐Cre;GCaMP6f offspring of either sex between the ages of postnatal Day 8 (P8) and P12 were used for experiments. Genotyping was accomplished using PCR.

### 2.2 Slice preparation

Transverse brainstem slices (200 μm thick) were prepared from P8–P12 mice. After rapid decapitation, the brainstem was removed from the skull and immersed in ice‐cold artificial cerebrospinal fluid (aCSF) containing (in mM): 125 NaCl, 2.5 KCl, 3 MgCl_2_, 0.1 CaCl_2_, 25 glucose, 25 NaHCO_3_, 1.25 NaH_2_PO_4_, 0.4 ascorbic acid, 3 myo‐inositol, and 2 Na‐pyruvate, pH 7.3–7.4, when bubbled with carbogen (95% O_2_, 5% CO_2_; osmolality of 310–320 mOsm/kg water). After cutting, slices were incubated in recovery aCSF bubbled with carbogen at 35°C for 30 min, and thereafter at room temperature.

### 2.3 Immunohistochemistry

Brainstem slices (100 μm thick) were cut and subsequently fixed with 4% (wt/vol) paraformaldehyde in phosphate buffer solution (PBS) for 20 min. Free‐floating sections were blocked in 4% goat serum and 0.3% Triton X‐100 in PBS for 1 hr. Slices were incubated with antibodies for chicken anti‐green fluorescent protein (GFP; 1:300; Abcam, Cambridge, UK, Cat# ab13970, RRID:AB_300798), rabbit anti‐oligodendrocyte transcription factor 2 (Olig2; 1:200; Abcam, Cambridge, UK, Cat# ab109186, RRID:AB_10861310), mouse anti‐neuronal nuclei (NeuN; 1:600; Millipore, Burlington, MA, USA, Cat# MAB377, RRID:AB_2298772) overnight at 4°C and subsequently incubated with different Alexa dye–conjugated secondary antibodies (1:500, Invitrogen, Carlsbad, CA, USA) for 2 hr at room temperature. Slices were mounted onto Superfrost slides in photobleaching‐protective medium (Fluoroshield; Sigma‐Aldrich, St. Louis, MO, USA). Stained slices were viewed with laser lines at 488, 555, and 633 nm using a 40× oil‐immersion objective on a confocal laser‐scanning microscope (LSM‐510, Zeiss, Oberkochen, Germany). Stack images were acquired at a digital size of 1,024 × 1,024 pixels with optical section separation (z interval) of 0.5 μm and were later cropped to the relevant part of the field without changing the resolution.

### 2.4 Ca^2+^ imaging

Ca^2+^ imaging was performed in normal aCSF at room temperature (22–24°C). Normal aCSF was the same as slicing aCSF, but with 1 mM MgCl_2_ and 2 mM CaCl_2_. Fluorescence images were acquired at 20 Hz sampling frequency using an ORCA Flash4.0 CMOS camera (Hamamatsu Photonics, Hamamatsu, Japan) and HCImage software (Hamamatsu Photonics, Hamamatsu, Japan), using wide‐field fluorescence microscope illumination (X‐Cite 120Q excitation light source with 120‐watt lamp). 1 mM glutamate (Sigma, St. Louis, MO, USA) or 40 mM KCl (Sigma, St. Louis, MO, USA) was puff‐applied through a glass pipette (5–7 psi for ~1 s), the tip of which was placed 50–100 μm away from the cell body. GCaMP6f‐GFP or Fluo‐4 (50 μM, Life Technologies, Carlsbad, CA, USA) fluorescence was measured when excited at 488 nm. Background subtractions were done for cells filled with Fluo‐4. Fluorescent regions of interest (ROIs) were defined manually. All values are expressed as a change in the fluorescence calculated as Δ*F*/*F*, where *F* is the basal fluorescence of the ROI and Δ*F* is the change in fluorescence of the ROI at the peak response relative to the *F* value. 6‐cyano‐7‐nitroquinoxaline‐2,3‐dione (CNQX; 50 μM; Tocris, Bristol, UK), 1‐naphthyl acetyl spermine (Naspm; 50 μM; Tocris, Bristol, UK), D‐AP5 (50 μM; Tocris, Bristol, UK), dantrolene (20 μM; Tocris, Bristol, UK), CdCl_2_ (200 μM; Sigma St. Louis, MO, USA), 4‐aminopyridine (4‐AP; 2 mM; Sigma, St. Louis, MO, USA), tertraethylammonium (TEA; 10 mM; Sigma, St. Louis, MO, USA), BaCl_2_ (1 mM; Sigma, St. Louis, MO, USA), tetrodotoxin (TTX; 1 μM; Sigma, St. Louis, MO, USA), nifedipine (100 μM; Sigma, St. Louis, MO, USA), and ω‐agatoxin‐IVA (agatoxin; 200 nM; Alomone, Jerusalem, Israel) were bath‐applied.

### 2.5 Electrophysiology

Whole‐cell patch‐clamp recordings were performed in normal aCSF at room temperature (22–24°C) using the voltage‐ or current‐clamp mode of the EPC‐10 (HEKA Electronik, Lambrecht/Pfalz, Germany). For Ca^2+^ current recordings, CsCl‐based pipette solution included (in mM): 150 CsCl, 10 TEA‐Cl, 1 MgCl_2_, 10 HEPES, 2 ATP, 0.3 GTP, 10 phosphocreatine, and 50 μM Fluo‐4, pH = 7.3. Ca^2+^ currents were analyzed after leak subtraction using a “traditional” p/4 stimulus train in EPC10‐Patchmaster. For current clamp and AMPAR current recordings, K‐gluconate‐based pipette solution contained (in mM): 130 K‐gluconate, 20 KCl, 5 Na_2_‐phosphocreatine, 10 HEPES, 4 Mg‐ATP, 0.3 GTP, and 50 μM Fluo‐4, pH = 7.3. Occasionally, pipette solution additionally contained Alexa 568 (50 μM) to visualize the cell through dye‐filling during whole‐cell recordings. Patch electrodes had resistances of 4–6 MΩ, and the series resistance (*R*_s_) was <25 MΩ before compensation.

### 2.6 Data analysis

Ca^2+^ imaging and electrophysiology data were analyzed and presented using Igor Pro (Wavemetrics, Lake Oswego, OR, USA; RRID: SCR_000325). Statistical significance was determined using paired Student's *t* test (GraphPad Prism, San Diego, CA, USA; RRID: SCR_002798). *p* Values < .05 were considered significant. Data values are reported as means ± standard error of the mean (*SEM*).

## 3 RESULTS

### 3.1 Glutamate‐induced Ca^2+^ rise mediated by AMPA receptors on pre‐myelinating OLs

**Figure 1:**
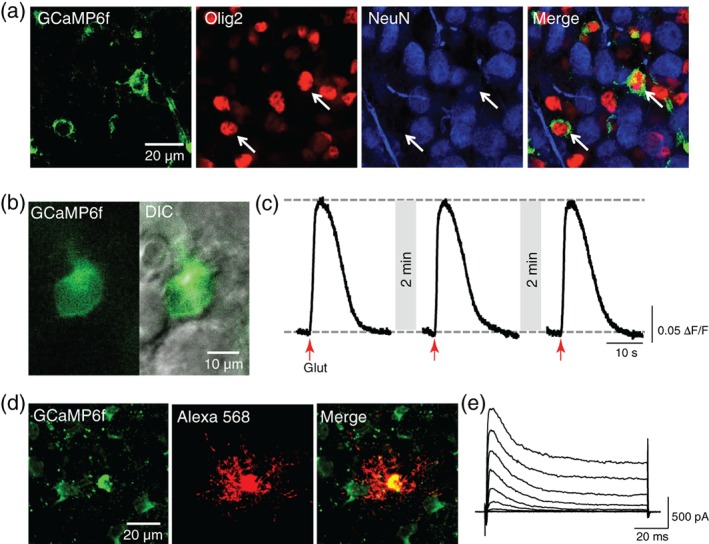
Glutamate induces Ca^2+^ rise in pre‐myelinating OLs. (a) GCaMP6f‐expressing cells (green, arrows) express OL marker Olig2 (red). Neuronal marker NeuN (blue) is expressed in nearby neurons, but not in GCaMP6f + OLs. (b) Fluorescent (left) and merge of DIC and fluorescent images (right) of a GCaMP6f + OL. (c) The cell responds to consecutive glutamate puffs (red arrow) with consistent Ca^2+^ rises. Two‐minute time intervals allowed for the recovery of the Ca^2+^ response to baseline. (d) A GCaMP6f+ cell (green) was dye‐filled with Alexa 568 (red) during whole‐cell patch‐clamp recording to discern its morphology. (e) Voltage‐activated currents in response to depolarization steps (from −80 to +50 mV, holding at −70 mV) in a GCaMP6f+ cell. OLs, oligodendrocytes [Color figure can be viewed at wileyonlinelibrary.com]

In the MNTB of the mouse auditory brainstem, GCaMP6f was expressed specifically in OLs, which were Olig2+ and NeuN‐, (Figure 1a) and were distinguishable under DIC and fluorescent imaging at 488 nm (Figure 1b). GCaMP6f + cells did not express PDGFRα, a marker of oligodendrocyte precursor cells (OPCs), but were positive for CC1, indicating that they are beyond the OPC stage (data not shown; Jang, Gould, Xu, Kim, & Kim, 2019). At P8‐P12, there is little compact myelin in the brainstem, and thus the majority of GCaMP6f + cells are presumed to be in the pre‐myelinating stage.

GCaMP6f was primarily detectable in the cytosol of OL cell bodies. Transient and local glutamate puff (1 mM) application 50–100 μm from the OL cell body resulted in a Ca^2+^ rise, as recorded by a transient increase in fluorescence (Figure 1c). The average amplitude of the Ca^2+^ response was 0.19 ± 0.01 Δ*F*/*F* (*n* = 113) and the decay time from the peak of the Ca^2+^ response to the resting Ca^2+^ level was 13.92 ± 0.52 s in 113 individual cells from 19 animals. Glutamate‐induced Ca^2+^ rise in OLs was replicable and consistent in amplitude after a two‐minute recovery period. Morphologically, the processes of GCaMP6f + cells did not align with axons to generate myelin sheaths (Figure 1d). In whole‐cell voltage‐clamp recordings, these GCaMP6f + cells displayed outwardly rectifying K^+^ currents and, and 37.5% of cells showed transient inward Na^+^ currents (Figure 1e). This electrophysiological profile aligns with what has previously been observed in pre‐myelinating OLs in the MNTB (Berret et al., 2017).

**Figure 2:**
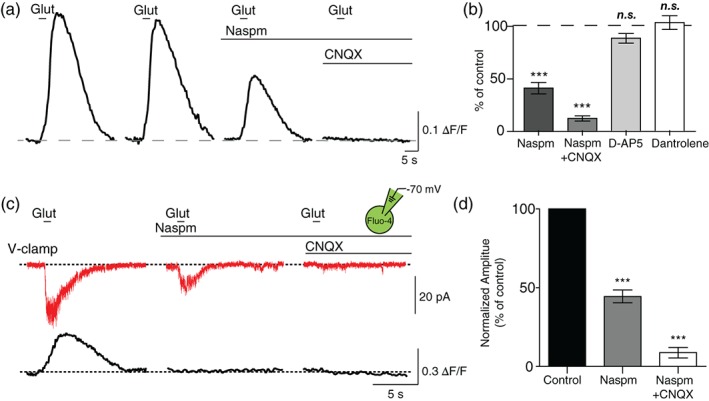
Ca^2+^ influx through Ca^2+^‐permeable AMPARs contributes to glutamate‐induced OL Ca^2+^ rise. (a) Ca^2+^ response to glutamate puff in a GCamP6f + OL in the presence of 50 μM Naspm and 50 μM CNQX to inhibit AMPARs. (b) Summary of Ca^2+^ responses in the presence of Naspm, Naspm, and CNQX, 50 μM D‐AP5, or 20 μM dantrolene. (c) Voltage clamp (V‐clamp) recordings of AMPAR‐mediated currents (red trace) in a single OL (at −70 mV) and associated Ca^2+^ rise (black trace) measured with Fluo‐4 in response to glutamate puff in control and the presence of Naspm and CNQX. (d) Summary of AMPAR‐mediated current amplitude in the presence of Naspm or Naspm and CNQX normalized to the control response. Data are represented as ± *SEM*. ** represents *p* < .01, and *** represents *p* < .001. OL, oligodendrocyte [Color figure can be viewed at wileyonlinelibrary.com]

OL lineage cells express ionotropic glutamate receptors (e.g., AMPA and NMDA receptors) and receive glutamatergic input from neurons (Berret et al., 2017; Micu et al., 2016). We tested whether AMPA and NMDA receptors are involved in glutamate‐induced Ca^2+^ transients in OLs using their antagonists. Before drug application, we confirmed the reliability of a Ca^2+^ response by verifying consistent Ca^2+^ rises in the OL in response to two consecutive glutamate puffs. The individual Ca^2+^ responses in the presence of antagonists were normalized to this control response. In the presence of Naspm, a Ca^2+^‐permeable AMPAR antagonist, the amplitude of glutamate‐induced Ca^2+^ transient was significantly decreased to 40.8 ± 5.46% of the control response from the same cell (*n* = 13 cells from four animals; *p* < .0001, paired *t* test; Figure 2a,b). Furthermore, when CNQX, an AMPAR antagonist, was added to the bath along with Naspm, glutamate‐induced Ca^2+^ rise was almost completely inhibited (11.8 ± 2.45% of the control response from the same cell; *n =* 24 cells from six animals; *p* < .0001, paired *t* test). We tested whether NMDA receptor activation contributes to glutamate‐induced Ca^2+^ rise in OLs. D‐AP5, an NMDA receptor antagonist, had no significant effect on glutamate‐induced Ca^2+^ rise in OLs (93.8 ± 3.99% of the control response from the same cell; *n =* 15 cells from three animals; *p* = .1398, paired *t* test). Other studies have shown the presence of Ca^2+^‐induced Ca^2+^ release (CICR) in OLs (Haberlandt et al., 2011; Ruiz, Matute, & Alberdi, 2010). However, dantrolene to block CICR had no significant effect on Ca^2+^ rise in OLs in response to glutamate puff (103.4 ± 6.55% of the control response from the same cell; *n =* 11 cells from three animals; *p* = .6144, paired *t* test).

To examine whether glutamate puff directly activates OL AMPARs to produce AMPAR currents, we recorded AMPAR currents and simultaneously measured Ca^2+^ rise in OLs. Fluo‐4, a Ca^2+^ indicator, was administered in the patch pipette during whole‐cell recording. In voltage‐clamp recordings (holding at −70 mV), the amplitude of AMPAR currents was −36.3 ± 6.04 pA in response to glutamate puff, which simultaneously induced a Ca^2+^ rise (*n =* 8 cells from seven animals; Figure 2c). Addition of Naspm to block Ca^2+^‐permeable AMPARs significantly reduced the amplitude of the AMPAR current to 44.5 ± 4.05% of control currents in the same cell (*n =* 8 cells from seven animals; *p* < .0001, paired *t* test; Figure 2d). CNQX application in addition to Naspm further inhibited the AMPAR current (8.8 ± 3.345% of control responses; *n =* 8 cells from seven animals; *p* < .0001, paired *t* test), indicating that glutamate induced AMPAR currents through both Ca^2+^‐permeable and ‐impermeable AMPARs in OLs. Interestingly, the Ca^2+^ rise in response to glutamate puff was completely inhibited in the presence of Naspm (1.3 ± 2.30% of control; *p* < .0001, paired *t* test). Thus, Ca^2+^ influx through Ca^2+^‐permeable AMPARs is mainly responsible for the Ca^2+^ rise when the membrane voltage was clamped at −70 mV. Notably, during GCaMP6f imaging when the membrane potential was not clamped, there was a significant portion of the Ca^2+^ rise remaining in the presence of Naspm, which was completely inhibited by CNQX (Figure 2a,b). These results indicate that AMPAR currents through both Ca^2+^‐permeable and ‐impermeable AMPAR activation contribute to glutamate‐induced Ca^2+^ response in developing OLs in the MNTB.

### 3.2 Voltage‐gated Ca^2+^ channels are a source for Ca^2+^ influx mediated by OL depolarization

**Figure 3:**
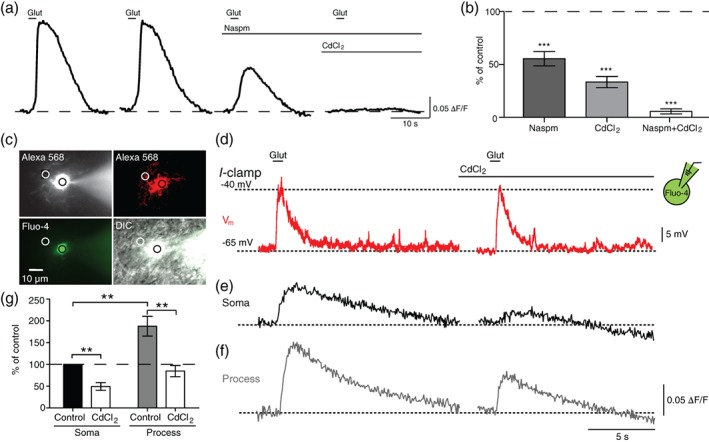
Voltage‐gated Ca^2+^ channels activated by depolarization contribute to Ca^2+^ rises in OLs. (a) Glutamate‐induced Ca^2+^ responses from a GCaMP6f + OL in the presence of 50 μM Naspm and additional 200 μM CdCl_2_. A horizontal line indicates baseline Ca^2+^ level. (b) Summary of Ca^2+^ response after the application of Naspm, CdCl_2_, or both. (c) Fluorescent image during whole‐cell recording (top left) of OL filled with Alexa 568, corresponding confocal image after fixation (top right), the fluorescent image of Ca^2+^ indicator dye Fluo‐4 (50 μM, bottom left), and merge of DIC and fluorescent images (bottom right) during whole‐cell patch‐clamp recording. Circles indicate the ROIs taken for the fluorescence intensity analysis. (d) Current clamp (*I*‐clamp) recordings of membrane voltage (V_m_, red trace) changes in OL in response to glutamate puff in the absence and presence of CdCl_2_. Glutamate puff depolarized OL to −40 mV from −65 mV. Note, CdCl_2_ had no effect on glutamate‐induced depolarization. (e, f) Subcellular Ca^2+^ responses of the same OL at the soma (e) and process (f) to glutamate puff correlated with depolarization in the absence or presence of CdCl_2_. (g) Summary of Ca^2+^ rise at soma and process in control and in the presence of CdCl_2_, which were normalized to control response in the soma. Data are represented as ± *SEM*. ** represents *p* < .01, and *** represents *p* < .001. OL, oligodendrocyte [Color figure can be viewed at wileyonlinelibrary.com]

How do Ca^2+^‐impermeable AMPAR currents contribute to Ca^2+^ responses in OLs? Our previous study showed that glutamatergic input depolarized pre‐myelinating OLs and consequently activated voltage‐gated ion channels (Berret et al., 2017). We examined whether Ca^2+^ influx via Ca_v_ channels could be a source of glutamate‐induced Ca^2+^ rise in OLs. After two consecutive applications of glutamate to confirm a replicable glutamate‐induced Ca^2+^ response, Naspm was bath applied to rule out Ca^2+^‐permeable AMPAR‐induced Ca^2+^ rise, reducing the amplitude of the Ca^2+^ response to 55.4 ± 6.70% of the control response from the same cell (*n =* 16 cells from four animals; *p* < .0001, paired *t* test; Figure 3a). The additional application of CdCl_2_, which blocks Ca_v_ channels, reduced the amplitude of the remaining Ca^2+^ response to 5.7 ± 2.34% of the control response from the same cell (*n =* 26 cells from four animals; *p* < .0001, paired *t* test, Figure 3b). CdCl_2_ alone reduced the amplitude of the Ca^2+^ response to 33.4 ± 5.31% of the control response from the same cell (*n =* 10 cells from three animals; *p* < .0001, paired *t* test). This result indicates that glutamate triggers Ca^2+^ entry through Ca_v_ channels in pre‐myelinating OLs.

Next, we examined whether glutamate application is sufficient to depolarize OL and consequently activate Ca_v_ channels. To study the correlation between glutamate‐induced depolarization and Ca^2+^ influx via Ca_v_ channel in pre‐myelinating OLs, we recorded membrane potential changes and simultaneously monitored a Ca^2+^ transient from a single OL (Figure 3c). In whole‐cell recordings of OLs with Ca^2+^ indicator Fluo‐4, glutamate puff elicited a depolarization from −65 mV to ~ − 40 mV (20.8 ± 2.00 mV change, *n =* 6; Figure 3d). Glutamate‐induced depolarization resulted in a Ca^2+^ rise (0.12 ± 0.021 Δ*F*/*F*, *n =* 6 cells from four animals; Figure 3e). By adding Fluo‐4 and Alexa 568 to the internal pipette solution, OL processes within 20 μm from the OL soma, which were not myelin sheaths, were clearly visible and showed detectable glutamate‐induced Ca^2+^ responses. The Ca^2+^ rise in the processes was 187.6 ± 22.76% of the response in the soma of the same cell (*n =* 6 cells from four animals; *p* = .0085, paired *t* test; Figure 3f). In the presence of CdCl_2_, the Ca^2+^ rise was significantly reduced in both processes and soma (*n =* 6 cells from four animals; *p* = .0012 for soma, *p* = .0018 for process, paired *t* test; Figure 3g), while glutamate‐induced depolarization was unaffected, indicating that glutamate puff is able to depolarize pre‐myelinating OLs and consequently activate Ca_v_ channels.

### 3.3 Increased neuronal activity increases extracellular K^+^ and glutamate, resulting in OL Ca^2+^ rise

**Figure 4:**
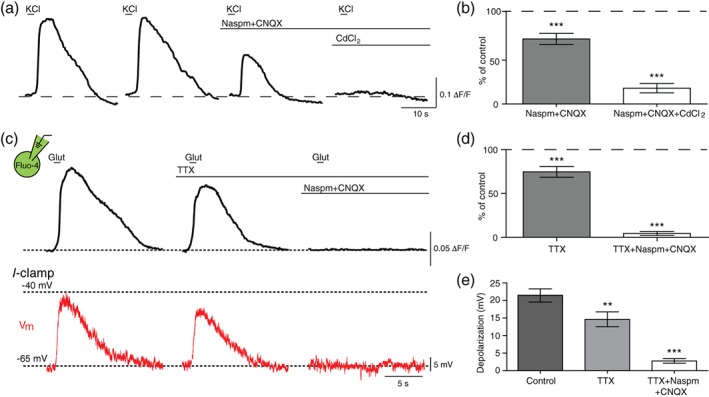
OLs depolarize in response to increased extracellular K^+^ and/or glutamate when neuronal activity increases. (a) Ca^2+^ responses to KCl puff (40 mM) in GCaMP6f + OL in the presence of Naspm and CNQX, and addition of CdCl_2_. (b) Summary of OL Ca^2+^ response to KCl puff in the presence of Naspm and CNQX, and the addition of CdCl_2_. (c) Ca^2+^ rise (black) as measured by Fluo‐4 and associated membrane voltage (*V*_m_) change (red) of OL in current‐clamp (*I*‐clamp) recordings in response to glutamate puff in the presence of 1 μM TTX, and addition of Naspm and CNQX. (d, e) Summary of glutamate‐induced Ca^2+^ responses (d) and depolarization (e) in OLs in the presence of TTX, Naspm, and CNQX. Data are represented as ± *SEM*. ** represents *p* < .01, and *** represents *p* < .001. OLs, oligodendrocytes; TTX, tetrodotoxin [Color figure can be viewed at wileyonlinelibrary.com]

During increased neuronal activity, K^+^ accumulates in the extracellular space and can depolarize OLs (Battefeld, Klooster, & Kole, 2016; Larson et al., 2018). We next tested the capacity of OL depolarization to recruit Ca_v_ channels allowing Ca^2+^ influx independent of OL AMPAR activation. KCl puff applied to OLs and surrounding tissue, which mimics elevated extracellular K^+^ when neuronal activity increases, resulted in a Ca^2+^ rise in GCaMP6f + OLs (Figure 4a). In the presence of Naspm and CNQX to isolate the OL depolarization‐induced Ca^2+^ entry, KCl‐induced Ca^2+^ rise was 70.5 ± 0.06% of control responses (*n =* 10 cells from three animals; *p* = .0008; paired *t* test). This remaining Ca^2+^ response was significantly reduced by the application of CdCl_2_ reduced to 24.3 ± 6.30% of responses in the presence of CNQX and Naspm (*n =* 10 cells from three animals; *p* < .0001, paired *t* test; Figure 4b). Together, these data indicate that OL depolarization is sufficient for Ca^2+^ influx via Ca_v_ channels.

We next determined whether increased neuronal activity and elevated extracellular K^+^ as a result of glutamate puff induced OL depolarization or whether AMPAR‐mediated currents directly depolarized OLs, or whether both mechanisms contributed. Using TTX to inhibit neuronal activity, we isolated AMPAR activation‐mediated OL depolarization. In whole‐cell current‐clamp recordings with Fluo‐4 in the intracellular pipette solution, glutamate puff induced a Ca^2+^ rise in the presence of TTX (1 μM), although TTX application resulted in a decrease in the Ca^2+^ rise in the presence of TTX to 74.7 ± 6.04% of control responses (*n =* 17 cells from five animals; *p* = .0007, paired *t* test; Figure 4c,d). Simultaneously, glutamate puff still depolarized OLs by 14.64 ± 1.13 mV under the inhibition of neuronal activity with TTX, which was a slight decrease from control (depolarization was 21.44 ± 1.88 mV in control; *n =* 6 from three animals; *p* = .0021; paired *t* test; Figure 4c,e). The reduction in the presence of TTX is assumed to be caused by the inhibited neuronal activity, although voltage‐activated Na^+^ channels in OLs could be also involved in the TTX‐sensitive response (Berret et al., 2017). The addition of Naspm and CNQX blocked the remaining Ca^2+^ response (4.5 ± 2.26% of control response; *n =* 17 cells from five animals; *p* < .0001, paired *t* test) and depolarization (2.8 ± 0.64 mV; *n =* 6 cells from three animals; *p* < .0001, paired *t* test). These results suggest that elevated extracellular K^+^ due to increased firing rate of neurons partially contributes to depolarization and Ca^2+^ rise in OLs, and that the larger remaining response is due to AMPAR‐mediated currents.

### 3.4 OL Ca^2+^ rise through P/Q‐type and L‐type Ca_v_ channels

**Figure 5:**
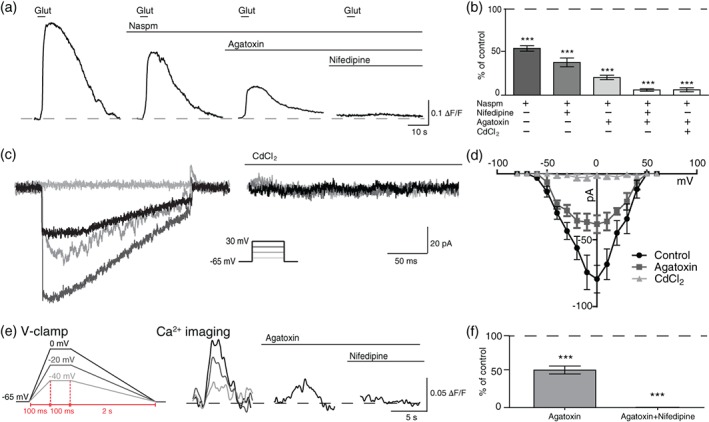
P/Q‐ and L‐type Ca_v_ channels mediated Ca^2+^ influx in response to glutamate input. (a) Glutamate‐induced Ca^2+^ rise in a GCaMP6f + OL in the presence of Naspm, agatoxin (200 nM) and nifedipine (100 μM). (b) Summary of Ca^2+^ responses after application of nifedipine or agatoxin, and combinations of agatoxin + nifedipine and agatoxin + CdCl_2_. (c) Ca_v_ channel currents in the presence of 4‐AP (2 mM), TEA (10 mM), BaCl_2_ (1 mM), and TTX (1 μM) in voltage‐clamp recording (holding at −65 mV), which were inhibited by CdCl_2_. Inset, step‐like depolarization protocol. (d) Current–voltage (I‐V) relationship of OL Ca^2+^ current (*I*_Ca_). *I*_Ca_ was partially inhibited by agatoxin and completely inhibited by CdCl_2_. (e) Ca^2+^ rise in a GCaMP6f + OL when directly depolarized using a depolarizing pulse (diagram) during voltage‐clamp recording in the presence of agatoxin and nifedipine. The OL was brought from resting membrane potential at −65 mV to −40, −20, or 0 mV over the course of 100 ms, held at 0 mV for 100 ms, and brought back to resting membrane potential over a period of 2 s. (f) Summary of depolarization‐induced Ca^2+^ rise in the presence of agatoxin and nifedipine. Data are represented as ± *SEM*. *** represents *p* < .001. OL, oligodendrocyte; TEA, tertraethylammonium; TTX, tetrodotoxin [Color figure can be viewed at wileyonlinelibrary.com]

Furthermore, we identified the types of Ca_v_ channels involved in Ca^2+^ influx in OLs in response to glutamate puff. We specifically examined L‐type and P/Q‐type channels, which are expressed in OL lineage cells (Cheli et al., 2015, 2016; Paez et al., 2009; Zhang et al., 2014). After Ca^2+^‐permeable AMPARs were blocked with Naspm, nifedipine (100 μM), an L‐type Ca_v_ channel blocker, reduced the glutamate‐induced Ca^2+^ response in GCaMP6f + OLs to 38.0 ± 5.09% (*n =* 18 cells from four animals; *p* < .0001, paired *t* tests), while agatoxin (200 nM), a P/Q‐type Ca_v_ channel blocker, reduced the amplitude to 20.6 ± 2.61% of the control response from the same cell (*n =* 25 cells from four animals; *p* < .0001, paired *t* tests; Figure 5a,b). Combined, agatoxin and nifedipine nearly abolished the glutamate‐induced Ca^2+^ rise, reducing the amplitude to 6.2 ± 1.33% of control responses (*n =* 10 cells from three animals; *p* < .0001, paired *t* test). The combination of agatoxin and CdCl_2_ reduced the Ca^2+^‐impermeable AMPAR‐mediated Ca^2+^ response to 6.4 ± 2.26% of control responses in the same cell (*n =* 10 cells from three animals; *p* < .0001, paired *t* test). Thus, P/Q‐type and L‐type Ca_v_ channels play a significant role in OL Ca^2+^ rise in response to glutamate puff.

To confirm the presence of functional P/Q‐type Ca_v_ channel currents in OLs, voltage‐activated Ca^2+^ currents (*I*_Ca_) were elicited from OLs in whole‐cell voltage‐clamp recordings. *I*_Ca_ was shown in response to depolarization steps from −80 mV to 60 mV and was completely inhibited by CdCl_2_ (Figure 5c). Partial Ca_v_ channel inactivation was observed, as expected for P/Q‐type Ca_v_ channels. Moreover, a portion of *I*_Ca_ was inhibited by agatoxin, suggesting that P/Q‐type Ca_v_ channels are involved in Ca^2+^ influx due to depolarization (Figure 5d). The voltage dependency of OL *I*_Ca_ was determined by plotting the current–voltage (*I*‐V) relationship curve. The peak of *I*_Ca_ at 0 mV was −79.86 ± 10.47 pA (n = 14 cells from eight animals). The presence of *I*_Ca_ and effect of Ca_v_ channel antagonists on glutamate‐induced Ca^2+^ responses demonstrates that Ca^2+^ influx via Ca_v_ channels importantly contributes to Ca^2+^ rise in pre‐myelinating OLs during glutamate‐induced depolarization.

We next tested the ability of direct OL depolarization to induce OL Ca^2+^ response mediated by P/Q‐type and L‐type Ca_v_ channels using whole‐cell voltage‐clamp. A depolarization pulse, mimicking the time course of the depolarization recorded in response to glutamate puff, resulted in a Ca^2+^ rise measured with GCaMP6f fluorescence. The Ca^2+^ rise was detectable in response to ~25 mV depolarization from −65 mV to −40 mV and was partially blocked by agatoxin application (52.3 ± 5.60% of control responses; *n =* 7 cells from four animals; *p* < .0001; paired *t* test; Figure 5e,f). The remaining Ca^2+^ response was inhibited by the addition of nifedipine (0.37 ± 0.08% of control responses; *n =* 7 cells from four animals; *p* < .0001; paired *t* test; Figure 5e,f), indicating that Ca^2+^ influx through a combination of P/Q‐type and L‐type Ca_v_ channels occurs in response to depolarization of OLs.

### 3.5 Axon stimulation provides sufficient glutamate for OL Ca^2+^ rise

**Figure 6:**
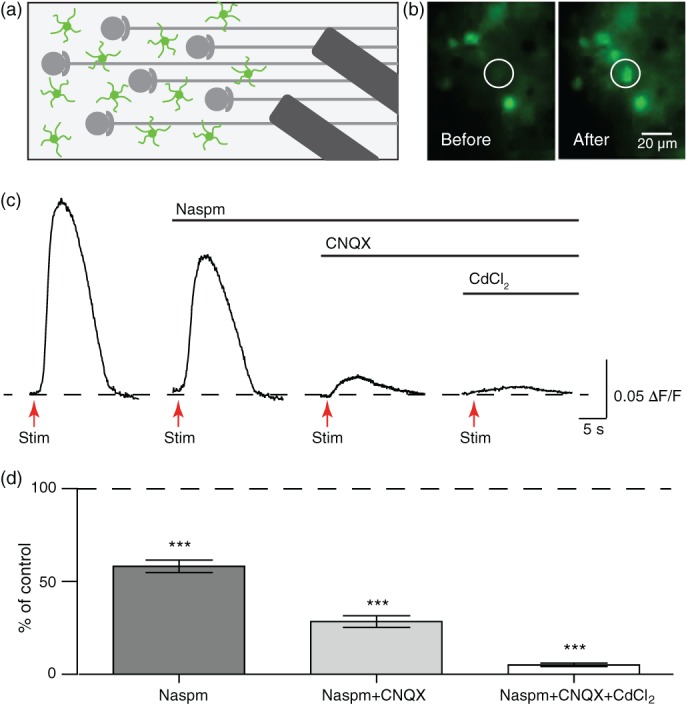
Axons provide sufficient glutamate to induce OL Ca^2+^ response. (a) Schematic of the experimental setup. A bipolar electrode was placed ~200 μm away from the MNTB to stimulate axon fibers (grey), and Ca^2+^ responses were recorded in GCaMP6f + OLs (green). (b) Fluorescent image of GCaMP6f + OL (white circle) before (left) and after (right) axon fiber stimulation at 50 Hz for 10 s. Note, some GCaMP6f + OLs did not respond to axonal stimulation. (c) OL Ca^2+^ response to axon fiber stimulation (red arrows) in the presence of Naspm, CNQX, and CdCl_2_. (d) Summary of Ca^2+^ rise in OLs in response to axon stimulation in the presence of Naspm, CNQX, and CdCl_2_. Data are represented as ± *SEM*. *** represents *p* < .001. MNTB, medial nucleus of the trapezoid body; OL, oligodendrocyte [Color figure can be viewed at wileyonlinelibrary.com]

We next examined whether neuronal activity causes glutamate‐mediated Ca^2+^ rise in OLs through neuron‐OL interaction. Axon fibers that innervate the MNTB were stimulated at 50 Hz for 10 s, inducing Ca^2+^ rise from GCaMP6f + OLs (Figure 6a,b). Blocking Ca^2+^‐permeable AMPARs with Naspm reduced the Ca^2+^ response to axon fiber stimulation to 58.2 ± 3.36% (*n =* 20 cells from four animals; *p* < .0001, paired *t* tests), and the combination of Naspm and CNQX reduced the response to 28.4 ± 3.16% of control responses in the same cell (*n =* 20 cells from four animals; *p* < .0001, paired *t* tests; Figure 6c,d), indicating that neuronal activity provides sufficient glutamate to induce Ca^2+^ rise. The Ca^2+^ response, which was not blocked by Naspm and CNQX, was significantly inhibited by Ca_v_ channel blocker CdCl_2_ (5.1 ± 0.99% of control responses in the same cell, *n =* 10 cells from two animals; *p* < .0001, paired *t* test). During axon fiber stimulation, increased neuronal activity leads to the accumulation of extracellular K^+^ and consequently OL depolarization (Battefeld et al., 2016; Larson et al., 2018). Indeed, we demonstrated that increasing extracellular K^+^ concentration using KCl puff depolarized and induced Ca^2+^ influx through Ca_v_ channels in OLs without AMPAR activation (Figure 4a). These data reveal that, when neuronal activity‐induced Ca^2+^ rise in OLs is mediated by AMPARs and Ca_v_ channels as sources of Ca^2+^ influx.

## 4 DISCUSSION

During development, neuronal activity drives physiological responses in OL lineage cells that influence OL maturation and myelination. It is proposed that neuronal activity triggers release of signaling factors to elicit OL Ca^2+^ transients and initiate downstream signaling processes. Here, we studied what factors mediate the dynamic communication between neurons and OLs leading to OL Ca^2+^ rise and the primary source of OL Ca^2+^ transients in response to neuronal input. The results reveal that increased neuronal activity leads to Ca^2+^ influx in OLs through glutamatergic signaling, mediated by AMPARs, depolarization, and L‐type and P/Q‐type Ca_v_ channels. These findings support that glutamatergic signaling is a key mechanism for neuron‐OL interaction that could lead to various downstream Ca^2+^ signaling pathways important for myelination and OL function in the mammalian nervous system.

Due to the overlap in expression of channels and receptors in both neurons and glia in the brain slice, it was difficult to isolate effects on single OLs and to quantify the contribution of each cell type to OL Ca^2+^ responses using pharmacological approaches. For example, bath application of CdCl_2_ can block axonal or presynaptic Ca^2+^ channels, inhibiting vesicular glutamate release, as well as oligodendroglial Ca^2+^ channels, and thus a mixed response could be observed. Glutamate puff could be activating AMPARs on neighboring cells, resulting in increased firing and release of other molecules or ions to trigger OL Ca^2+^ transients. During periods of increased action potential firing, the concentration of K^+^ in the extracellular area increases, which in turn depolarizes OLs (Battefeld et al., 2016; Larson et al., 2018). Our findings in Figure 4 showed that increased extracellular K^+^ release from neighboring cells partially contributes to OL Ca^2+^ transients. This depolarization could then activate Ca_v_ channels in OLs, resulting in a Ca^2+^ rise. In addition, ATP and adenosine are other possible molecules, which are released in response to axon stimulation and have been shown to induce a Ca^2+^ rise in OL lineage cells (Hamilton et al., 2008; Hamilton, Vayro, Wigley, & Butt, 2010; Stevens, Porta, Haak, & Fields, 2002). Activation of neighboring cells may impact OL Ca^2+^ transients and explain the slow and prolonged kinetics of the Ca^2+^ rise in OLs in response to electrical stimulation of axons seen in Figure 6. In physiological conditions, Ca^2+^ dynamics in OLs are a result of a summation of various inputs over time when neuronal activity increases.

In this study, we examined OL Ca^2+^ rise to delineate sources of Ca^2+^ influx in OLs in response to glutamatergic input. An alternative mediator of Ca^2+^ transient duration may be a secondary Ca^2+^ source, such as release from internal stores in OLs. Amplitude and duration of Ca^2+^ transients in OLs induced by AMPA application were reduced after blocking ryanodine receptors (Ruiz et al., 2010), implicating a role for CICR from internal stores in AMPAR‐mediated Ca^2+^ transients. We tested the effect of dantrolene, a blocker of CICR, on glutamate‐induced Ca^2+^ rise in OLs, but there was no significant effect (Figure 2). In hippocampal NG2+ cells, Haberlandt et al. (2011) demonstrated glutamate‐induced Ca^2+^ rise mediated by Ca_v_ channels and Ca^2+^‐permeable AMPARs, similarly observed in the present study. In contrast to our results, Ca^2+^ influx triggered CICR and additionally amplified Ca^2+^ rise in hippocampal NG2+ cells (Haberlandt et al., 2011). As another intracellular Ca^2+^ source, a recent study demonstrates that mitochondrial Ca^2+^ release mediates microdomain Ca^2+^ transients and large Ca^2+^ waves in myelin sheaths (Battefeld, Popovic, de Vries, & Kole, 2019). This suggests that, in myelin sheaths of mature OLs, mitochondrial Ca^2+^ regulation is important for myelin remodeling.

What is the functional relevance of Ca^2+^ rise in OLs in response to glutamatergic input? Glutamate has been repeatedly shown to induce pathological damage to cultured OLs (Alberdi, Sánchez‐Gómez, Marino, & Matute, 2002). Similar to what occurs in neurons, excitotoxicity due to over‐activation of AMPARs leads to Ca^2+^ rise and downstream cascades leading to mitochondrial damage and apoptosis (Leuchtmann, Ratner, Vijitruth, Quab, & McDonald, 2003; Ruiz et al., 2010; Sánchez‐Gómez, Alberdi, Ibarretxe, Torre, & Matute, 2003). An irreversible elevation of Ca^2+^ through either AMPAR or Ca_v_ channels could induce necrosis of OLs in pathological conditions. However, dynamic Ca^2+^ transients are beneficial for signaling cascades of physiological processes of OLs, such as increased OL lineage cell survival and migration, as well as myelination (Gautier et al., 2015; Gudz, Komuro, & Macklin, 2006; Kougioumtzidou et al., 2017). Neuronal activity elicited a Ca^2+^ response in OLs in zebrafish, which was associated with myelin elongation or retraction, depending on the dynamics of the Ca^2+^ response (Baraban et al., 2018; Krasnow et al., 2018). In co‐cultures with dorsal root ganglion neurons, vesicular release of glutamate from axons stimulates local translation of myelin basic protein in OPCs and stimulates myelin induction through activation of NMDARs and mGluRs, rather than AMPARs (Wake et al., 2011, 2015). In the mouse brainstem, we identified AMPARs and Ca_v_ channels as an important Ca^2+^ influx pathway for Ca^2+^ dynamics in developing OLs. Recent studies suggest the potential role of AMPARs in OL development and myelination (Chen et al., 2018; Kougioumtzidou et al., 2017). Alterations in AMPAR expression in OPCs impact OL lineage cell differentiation, survival, and myelination in the corpus callosum (Chen et al., 2018; Kougioumtzidou et al., 2017). In addition, the electrical changes of the OL membrane are also important for OL development, because the knockdown of Ca_v_ channels or Na_v_ channels throughout the OL lineage impaired OL development and myelination (Berret et al., 2017; Cheli et al., 2015, 2016). These studies support that AMPARs and Ca_v_ channels, as well as other signaling mechanisms, can play a role in OL Ca^2+^ dynamics as important mediators of glutamate‐mediated signaling to impact OL development.

Dynamic interaction between active axons and OLs in the auditory brainstem is particularly relevant to auditory function, because this brain region is heavily myelinated and operates at the upper limits of action potential frequency and speed required for sound localization (Kim, Renden, & von Gersdorff, 2013; Kim, Turkington, et al., 2013; Sinclair et al., 2017). Still, neuronal glutamate‐induced Ca^2+^ rise in OLs has been observed in brain areas other than the MNTB. Neuronal activity induced myelination‐associated Ca^2+^ rises in OLs in the zebrafish spinal cord (Baraban et al., 2018; Krasnow et al., 2018). Furthermore, OPC Ca^2+^ rise in response to glutamate or depolarization has been observed in the rodent optic nerve and hippocampus (Haberlandt et al., 2011; Hamilton et al., 2010; Sun et al., 2016). The present study provides a mechanistic explanation for neuronal activity‐dependent Ca^2+^ rise in pre‐myelinating OLs in the MNTB, but could be relevant for Ca^2+^ transients in developing OLs in other brain areas. Understanding the physiological properties of OLs and mechanisms of neuron‐OL interaction throughout the OL lineage and throughout the brain is critical for further comprehension of their roles in brain function. The mechanism of Ca^2+^ rise in pre‐myelinating OLs resulting from depolarization and neuronal input demonstrated in this study provides important insight into the dynamic physiological properties of OLs.

## CONFLICT OF INTEREST

The authors declare no competing interests.

## AUTHOR CONTRIBUTIONS

T.B. performed all experiments, analyses, and drafted the manuscript. J.H.K. supervised the project and revised the manuscript. All authors reviewed and approved the final manuscript.
